# Disease causing poverty: adapting the Onyx and Bullen social capital measurement tool for China

**DOI:** 10.1186/s12889-020-8163-5

**Published:** 2020-01-14

**Authors:** Lizheng Xu, Min Guo, Stephen Nicholas, Long Sun, Fan Yang, Jian Wang

**Affiliations:** 1 Center for Health Economics Experiment and Public Policy, School of Public Health, Shandong University; Key Laboratory of Health Economics and Policy Research, NHFPC (Shandong University), Jinan, China. No.44 Wenhuaxi Road, Lixia District, Jinan, 250012 China; 2 Dong Fureng Institute of Economic and Social Development, Wuhan University, 54 Lishi Hutong, Dongcheng District, Beijing, 100010 China; 3 Top Education Institute, 1 Central Avenue, Australian Technology Park, Eveleigh, Sydney, NSW 2015 Australia; 4 Newcastle Business School, University of Newcastle, University Drive, Newcastle, NSW Australia; 5 Guangdong Research Institute for International Strategies, Guangdong University of Foreign Studies, 2 Baiyun North Avenue, Baiyun, Guangzhou, 510420 Guangdong China; 6 School of Management and School of Economics, Tianjin Normal University, West Bin Shui Avenue, Tianjin, 300074 China

**Keywords:** Social capital, Measurement tool, Onyx-Bullen scale, Disease-causing-poverty

## Abstract

**Background:**

Disease-causing poverty is a serious problem in rural China, where social capital can mediate the disease—poverty relationship. However, there is no generally accepted reliable, robust and viable measure of social capital for China’s unique socio-cultural context. This study adapts for China the widely used Onyx and Bullen social capital measurement scale and tests the validity and reliability of a modified Chinese Onyx-Bullen general scale, the Chinese Onyx-Bullen health scale, for a disease-causing-poverty subpopulation in rural China.

**Methods:**

We conducted the forward and backward translation procedure and cross-cultural adaptation process to derive the 34 item Chinese Onyx-Bullen general scale. Next we collected through face-to face interviews a sample of disease-causing poverty population in rural Shandong province in China to test a 29 item modified Chinese Onyx-Bullen general scale for a health subpopulation. Most of the rural respondents had no formal work, so 5 work-related items in the Onyx-Bullen general scale were deleted in the Chinese Onyx-Bullen health scale. Exploratory factor analysis was conducted to evaluate the structure, validity, internal consistency and reliability of the Chinese Onyx-Bullen health scale. SPSS21.0 software was used for data analysis.

**Results:**

A total of 467 people completed the scale. For the 29-item scale, a better simple structure was found when the number of factors was limited to 8. The absolute values of inter-factor correlations were in the range of 0.004 to 0.213 and the Kaiser-Meyer-Olkin value was 0.834. All the eight factors explain a total of 59.51% of the variance. The total scale had a Cronbach’s alpha = 0.868, in which seven of the eight factors had Cronbach’s α greater than 0.5.

**Conclusion:**

The Chinese health version of the Onyx-Bullen general social capital scale showed an adequate reliability and validity in a rural disease-causing poverty subpopulation in Shandong province, providing the first general, robust, consistent and reliable measure of social capital in China. The Chinese Onyx-Bullen general social capital scale provides a scale for testing social capital in China or for modification along the lines of the Chinese Onyx-Bullen health scale.

## Background

### Social capital, health and poverty

Social capital is a powerful concept informing social science and health research. With a heritage going back more than a century [1], the common thread that defines social capital is the idea of different types of social networks create shared resources, which are allocated, not always equally, among group members within obligatory sets of social, economic and political relationships [2–4]. Social networks funnel growth enhancing resources to network members, including capital, information and knowledge, that attenuates poverty or falling into poverty [5, 6]. Kawachi and Berkman identified eight fields of social inquiry that have examined the links between social capital and diverse outcomes: (1) families and youth behavior problems, (2) schooling and education, (3) community life, (4) work and organizations, (5) democracy and governance, (6) economic development, (7) criminology and (8) public health [7]. Research on social capital and public health has revealed the interaction between health, poverty and social capital. In the public health field, studies have utilized social capital to analyze the mechanisms that link social inequalities, both positively and negatively, to physical health [8–17]; mental well-being (depression, anxiety, stress); health risk behaviors (use/misuse of alcohol, smoking, illicit drugs, sexual behavior); behavioral issues (conduct problems, aggression/violence, delinquency); and self-rated health and quality of life (physical health, happiness, satisfaction with life) [18–21]. In each case, individuals and families that share norms, values and network, access (or are excluded from) resources that deliver (or deny) improved outcomes. Besides facilitating access to medical treatment, the type and quantity of resources, such as capital and credit, information about debt financing and borrowing, access to social services and employment opportunities, accessed through a household’s social ties both prevents poverty and facilitates recovery from poverty [5, 22, 23]. The poverty-social capital-health relationship has argued that poverty and income inequality erode social capital and breed poor health outcomes [24–26]. More recently, scholars have investigated how diseases cause poverty to families [27]. The concept of the “disease poverty trap”, which is also named iatrogenic poverty [28] or the disease-driven poverty trap [29], was developed to describe the disease-causing-poverty phenomenon. For example, Bruno reported that disease could lead to poverty by two major pathways: the death or disability of a household income earner due to disease, and high medical-related cost related to disease treatment [28]. Poverty caused by death or disability due to disease of the household income earner is measured by the official poverty line, defined as households that had a per capita income lower than RMB683 in China [23]. Poverty due to medical expenses is defined as out-of-pocket medical expenses in excess of 40% of household income, after any government health care subsidy, which reduces non-health household expenditure below the level required for necessities [30]. Margaret illustrated four effects of serious illness: untreated sickness, delayed health-care seeking, irrational use of drugs and extremely heavy health-care costs, where medical expenses tip households into poverty [27]. Matthew and his colleagues developed a general one-disease SIS model and concluded that cumulative infections have the potential to keep a population in a poverty trap [29]. Most governments recognise the challenge of disease related poverty, at least partially publicly funding their health sector, especially for the poor [28], to provide minimum levels of health care quality and access to health services. Faced by large medical expenses, social capital provides a partial safety net, shielding households from poverty [31].

Disease, social capital and poverty is a circular relationship, where social capital attenuates disease creating poverty and poverty creating disease. While many studies of inequality, health and social capital have focused on social capital from developed countries [32–36], the links between inequalities, social capital and health are particularly important in developing countries. Social capital can improve health outcomes by influencing access to health services and amenities, impacting health-related behaviours, and affecting individual psychosocial processes [7, 32, 37–42]. Lifting 800 million out of poverty between 1978 and 2018, China has reduced rural poverty from 97.5% of the rural population living in poverty in 1978 to only 3.1% in 2018 [43]. Today, 30 million people live in poverty in China [44], with 42% due to disease-causing-poverty [45]. At risk households include those already below or on the poverty line; with low educational attainment; depended on agricultural income especially in western rural provinces; with a high dependency ratio; with members in poor health (including disabilities), old aged and children; and with debts or no savings and few assets. In 2012, 12.9% of Chinese households were burdened with a “catastrophic” levels of health expenditures, putting families “at risk” of poverty [46]. But, studies of social capital health outcomes and poverty remain limited and inconclusive [47–49]. In China, research has mainly focused on the relationship between social capital and mental health [50–52], health related behaviors [53–55], income inequality [56] and special populations, such as pregnant women [57] AIDS patients [58, 59] domestic migrants [60] and diabetes patients [61]. Previous articles reporting on the relationship between social capital. Health and poverty in China have tended to focus on urban populations [62] and older people [63]. Researchers have warned that social capital displays rural-urban differences, and cultural and socioeconomic factors should be considered when using social capital measures [64].

Gaps in the health-poverty-social capital literature are compounded by the lack of a robust, commonly agreed and context-specific tool to measure social capital. Under different environmental, cultural and ethnic conditions, there are differences in the form, and therefore the measure, of social capital [39, 65]. Given its unique historical and cultural traditions, and a large population suffering from diseases at any one time, a context-specific and universally agreed measure of social capital is need for China [41, 66]. This paper proposes and tests such a social capital measure.

### Problem of social Capital measures

In China, the urgent need to investigate the social capital of vulnerable groups suffering poverty and disease is constrained by effective and comparable tools to measure social capital. Researchers [67, 68] have argued that existing social capital measures used in China depend on scales formed in other countries, self-designed scales and self-selected indicators related to social capital. With numerous approaches to measuring social capital, Agampodi et al. [48] pointed out that there has been no universally applicable gold standard tool to measure social capital, resulting in discrepancies, inconsistencies and disagreements between competing scales. Without a standard social capital measure, comparisons between different studies and different countries are problematic [31, 69].

In terms of previous research on social capital in China, different methods and social capital tools were used by researchers. Many social capital measures nonrandomly select specific items from existing social capital scales [52, 70], such as the World Bank’s SC Assessment Tool [50, 54]. Some researchers developed a social capital scale based on previous related studies [58, 60, 71, 72], while others used national or international large surveys, selecting social capital related items in pre-existing questionnaires [50, 73]. With no consensus around the social capital measuring tool, and specific social capital factors inconsistent across the various studies, the results from previous studies cannot be compared or integrated. In addition, some health studies were limited because they rely on secondary social capital data, which are rarely comparable with social capital measures specially designed for target populations [17]. Therefore, a professional, effective and universally applicable social capital tool is urgently need for China, especially to investigate the disease-poverty relationship. We adapt the existing and widely used Onyx and Bullen [18, 74] social capital scale for social capital research in China, applicable not only to health studies, but a diverse range of studies including social capital and climate change, environmental activism [75], community governance [76], small-medium enterprise knowledge management [77] and sport event participation [78].

The Onyx-Bullen scale was modified for the Chinese context—the Chinese Onyx-Bullen general scale—applicable to health and non-health research. We then made mainly minor further adaptations to the Chinese Onyx-Bullen general scale to measure social capital for disease causing poverty research, the Chinese Onyx-Bullen health scale. The Chinese Onyx and Bullen health social capital scale was tested for validity and reliability for disease causing poverty populations in rural China. Given the performance of the Chinese Onyx-Bullen health scale, we are confident that our Chinese Onyx-Bullen general scale is also robust, consistent and reliable for other populations, with and without further minor modifications.

### The scale

Designed to measure social capital across five different communities in Australia, the Onyx and Bullen [74] social capital scale defined the social capital concept; developed a valid and practical scale to measure social capital; and investigated the reliability and validity of the scale. Measured on a 4-point Likert-type response from 1 (no, not much or no, not at all) to 4 (yes, definitely or yes, frequently), the Onyx-Bullen scale comprised 36 items, with 8 specific independent factors: Participation in the Local Community, Proactivity in a Social Context, Feelings of Trust and Safety, Neighborhood Connections, Family and Friends Connections, Tolerance of Diversity, Value of Life and Work Connections. In their study, the overall reliability of the scale was good, with Cronbach’s alpha was 0.84 and inter-total correlations in the range of 0.25 to 0.45, confirming the psychometric strength of the original scale.

The Onyx-Bullen social capital scale has been tested and applied in other countries and regions. Raika Abdulahad [79] translated social capital scale into Arabic, investigating the social capital of Iraqi-Canadians, concluding that the translated instrument had adequate reliability and validity for this special population. Using a sample of 496 American respondents, O’Brien et al. [80] evaluated the Onyx-Bullen social capital scale, also confirming the modified scale as reliable and valid compared with Onyx and Bullen’s original research. Arezoo Yari et al. [81] translated the scale into Persian, tested it on medical students in Iran and concluded that the translated scale was appropriate for further use in social health research. The Greek version of the Onyx-Bullen scale was evaluated by Kritsotakis et al., establishing that the translated scale was useful to measure individual-level social capital in Greece [82]. Using 31-items from the Onyx-Bullen scale, Allison Webel et al. [83] assessed the social capital of people living with HIV/AIDS from five countries. To measure the health and health-related variables for the Australian population, Dean [84] used six items from Onyx-Bullen scale to measure perceptions of social cohesion and Joanne Allen [85] used nine items of Onyx-Bullen scale to assess the social capital condition of residents aged 55 and over from two communities in Australia. After testing and use in different countries, different subgroups within a country and diverse social-cultural contexts, the Onyx-Bullen scale has been shown to be adaptable, robust and reliable when translated into different languages.

## Method

### Scale translation and cross-cultural adaptation

Our large cross-sectional study is the first time the Onyx-Bullen scale has been adapted and validated for use in China. We received permission from Onyx and Bullen to translate, adapt and use their final 2000 scale. The translation of the Onyx-Bullen scale followed the standard forward and backward procedure. First, professors of public health and a graduate student majoring in English translated the original English scale into Chinese (forward step). Next, an English native speaker, a public health professor and a graduate student majoring in English back-translated from Chinese into English (backward step). The two English versions were compared, and modifications were made to guarantee the integrity of the question content and ensure a fluent final version. For example, subordinate clauses were put before the main clauses and adverbs were placed at the beginning of a sentences, to make them more suitable for Chinese reading and comprehension habits.

After the translation into Chinese, two Chinese professors and two Chinese graduates in public health undertook the task of cultural adaption. For example, the original question “Have you ever picked up other people’s rubbish in a public place?” was changed into “Have you ever help to pick up rubbish in a public place?”, to reveal respondents’ initiative in protecting the public environment. The question “If you were to die tomorrow, would you be satisfied with what your life has meant?” was not acceptable in rural China, where it would be considered impolite and taboo. The question was changed to “If you have only on day left in your life, are you satisfied with what your life has meant?”, to make the question more euphemistic and acceptable to rural respondents. Several of the Onyx-Bullen survey questions related to work content were not applicable to our rural poor people sample. For our rural study, we excluded 5 items in the Onyx-Bullen survey original scale, while retaining a question about paid employment. The general and health Onyx-Bullen scale are identical, except for 5 items related to specific types of paid employment. These 5 items in the Chinese Onyx-Bullen general scale were not validated for our rural sample. The final health scale comprised 29 items. After the translation and cultural adaption, researchers and a native speaker compared the translated Chinese scale and the original scale, and assessed its applicability to the Chinese rural environment to determine any final modifications. As a final check, the Chinese and English scales were read by a graduate student fluent in both Chinese and English to ensure all the essential Onyx-Bullen scale information was included in the translated general version.

### Sample and scale

The validation of a Chinese version of the health Onyx and Bullen social capital scale was undertaken using data from a large cross-sectional study investigating the health and welfare of disease-related rural households in poverty. Recruitment of interviewees focused on a specific disease-related poor subpopulation. The sample was derived from the Shandong Provincial Health Poverty Alleviation Information System, which contained data on all the disease-related poverty households in Shandong province. Inclusion criteria were households with a per capita income lower than RMB683 and suffering from one or more of 93 major serious diseases with high medical cost or inability to pay the hospitalization costs.

Stratified and random sampling were employed: in step one, 2 of 17 cities in Shandong province were selected; in step two, 14 townships in the 2 cities were selected; in step three, 41 rural village committees were selected; in step four, 20 households in each rural committee were selected; and finally one eligible household member was selected to be the survey respondent. A total of 802 eligible disease-causing poverty households were included in the investigation, with the interviewers meeting the following two conditions: being the patients or close family member caregivers of the patients, as some patients could not finish the questionnaire due to their physical condition; and knowing the household’s financial details.

After deleting missing values, among all the 802 interviewers, a total of 467 households completed the questionnaire, providing a response rate of 58.23%. The missing data were mainly due to our sample of rural, poorly educated and elderly people not able to understand all the social capital questions; their physical condition, including advanced age, mental disorders and disabilities, restricting their ability to answer the survey; or declining to give their informed written consent. Our 58% response rate was greater than the benchmark 50% considered adequate for similar research [86–88] and in other published research [89, 90]. While households that did not complete the survey might not be randomly distributed across disease-related poverty households in Shandong province, our large final sample size of 467 households gives confidence on the robustness of our findings.

In previous studies using the Onyx—Bullen scale, both workers and non-workers were included in the sample. Our translation continued the work—non-work connection, with work-related questions. Our sample comprised poor people, with diseases, living in rural areas, whose main source of their income was from farming and allowances. Therefore, the 5 work-related items in the Chinese Onyx-Bullen general scale were modified to one work-related item in the health Onyx-Bullen scale, and tested for our rural disease-causing poverty subpopulation.

### Data analysis

Using SPSS21.0 software, we performed exploratory factor analysis to determine the best factor structure in our data and then compared our results with those of Onyx and Bullen and other studies using the Onyx-Bullen survey. For exploratory factor extraction, principal components analysis was used. A better simple structure was found when the number of factors was limited to 8. Following Wu [91], both orthogonal rotation and oblique rotation were employed. We used Varimax and Direct Oblimin to observe the correlation between different factors and to determine the item-loading rotation method. Cronbach’s α measured internal consistency and reliability of the scale and each factor. When Cronbach’s α ≤ 0.6, the internal consistency was generally considered insufficient; 0.6 < Cronbach’s α ≤ 0.8 indicated the reliability was fair; 0.8 < Cronbach’s α ≤ 0.9 the scale was good reliability; and Cronbach’s α ≥ 0.9 showed that reliability was excellent [91].

## Results

### Sample description

Of the 467 final respondents, male respondents (248, 53.1%) were only slightly more numerous than female respondents (219, 46.9%). Respondents aged 60 and above, accounting for more than 70% of the total respondents. Almost 80% of the respondents only had a primary school education and below, among which 46.5% were without any education. In terms of self-reported health, 58.2% had one chronic or serious disease, with 35 respondents (7.5%) having three or more diseases. Most of the diseases were chronic diseases, with the four highest number of respondents were cardiovascular and cerebrovascular diseases, heart diseases, joint diseases and diabetes (see Table 1).

**Table 1:** Demographic Information

Variables	N	Frequency (%)
Gender		
Male	248	53.1%
Female	219	46.9%
Age		
≤ 49	47	10.1%
50–59	77	16.5%
60–69	133	28.5%
70–79	145	31.0%
80–89	60	12.8%
≥ 90	5	1.1%
Education		
None	217	46.5%
Primary school	151	32.3%
Middle school	81	17.3%
High school	17	3.6%
Specialized college	1	0.2%
Marital status		
Unmarried	34	7.3%
Married	307	65.7%
Separated or devoiced	10	2.1%
Widowed	114	24.4%
Number of disease		
None	65	13.9%
One	272	58.2%
Two	95	20.3%
Three or more	35	7.5%

## Factor analysis

Our adapted Onyx-Bullen health scale comprised 29 items modified for China’s social-cultural circumstances. Exploratory factor analysis was conducted to determine the load of the items and to which factor each item belonged. Inter-factor correlation was reflected in the factor correlation matrix (see Table 2). The direct oblique Oblimin rotation identified the extent of intercorrelation between the separate factors in the component correlation matrix. The absolute values of inter-factor correlations were in the range of 0.004 to 0.213, with all scores failing to reach to 0.3. As shown in Table 2, the factors are not highly correlated with each other and can be considered as independent. According to Nunnally and Bernstein [92], when the correlation is less than 0.3, compared to oblique rotation, the orthogonal varimax rotation method is better because the simple results of the factor pattern and factor structure is approximately the same while orthogonal gained the benefit of its simplicity. The result of factor analysis using orthogonal rotation method is shown in Additional file 1: Table S1. The Kaiser-Meyer-Olkin value was 0.834. Bartlett’s test χ^2^ was 4139.26 with df 406 (*p* < 0.000). These eight factors explain a total of 59.51% of the variance, in which the three factors explaining the variance most were factor 1 (11.94%, indicating *Participation in the Local Community*), factor 2 (9.95%, indicating *Feelings of Trust and Self-value*) and factor 3 (7.59%, indicating *Neighborhood Connections*).

**Table 2:** Social capital factors correlation matrix

Factors^a^	2	3	4	5	6	7	8
1	.188	.211	−.213	.187	−.120	−.105	.140
2		.141	−.134	−.008	.004	−.116	.111
3			−.121	.202	−.138	−.207	.208
4				−.182	.061	.125	−.053
5					−.096	−.198	.064
6						.075	−.045
7							−.129

^a^ Factor1 = Participation in the Local Community; Factor2 = Feelings of Trust and Self-value; Factor3 = Neighborhood Connections; Factor4 = Proactivity in a Social Context; Factor5 = Tolerance of Diversity; Factor6 = Voluntariness; Factor7 = Feelings of Safety; Factor8 = Friends Connections

The rotation of factor analysis converged after 12 iterations. As shown in Additional file 1: Table S1, 29 items were attributed to 8 factors. The meaning of each factor was identified from the item content. The factor referring to *Participation in the Local Community* included 6 items; the factor referring to *Feelings of Trust and Self-value* included 6 items; the factor referring to *Neighborhood Connections* included 5 items; the factor referring to *Proactivity in a Social Context* included 4 items; the factor referring to *Tolerance of Diversity* included 2 items; the factor referring *Voluntariness* included 2 items; the factor referring *Feelings of Safety* included 2 items; and the factor referring *Friends Connections* included 2 items. Factors were reclassified and explained under China’s socio-cultural conditions, becoming slightly different from the original factors in Onyx-Bullen scale.

### Reliability

The reliability coefficient—Cronbach’s alpha—of the total scale, as well as it of each factors, indicated internal consistency. Our 29 total-item social capital scale had a Cronbach’s alpha = 0.868 from the sample of 467 respondents. No item resulted in an increase in overall Cronbach’s α after deletion. The factor representing *Participation in the Local Community* had a Cronbach’s α = 0.829; the factor representing *Feelings of Trust and Self-value* had a Cronbach’s α = 0.758; the factor representing *Neighborhood Connections* had a Cronbach’s α = 0.665; the factor representing *Proactivity in a Social Context* had a Cronbach’s α = 0.682; the factor representing *Tolerance of Diversity* had a Cronbach’s α = 0.794; the factor representing *Voluntariness* had a Cronbach’s α = 0.490; the factor representing *Feelings of Safety* had a Cronbach’s α = 0.573; and the factor representing *Friends Connections* had a Cronbach’s α = 0.441. According to Wu [91], the reliability of the whole instrument would be acceptable if Cronbach’s coefficient α was 0.6 or greater, and the Cronbach’s coefficient α of each factor should be equal to 0.5 or greater. In our study, except *Voluntariness* and *Friends Connections*, all factors had Cronbach’s α greater than 0.5.

## Discussion

In health-poverty research in China, there is an urgent need for a robust sociocultural China-specific social capital scale. Used extensively for nearly 20 years, this study adapted and validated the Onyx--Bullen social capital scale for Chinese socioeconomic-cultural circumstances. We derived an Onyx-Bullen general scale for China, the further modified that scale for our sample of disease-related rural poverty households. The original 34-item Onyx and Bullen scale was translated into Chinese as the general scale, then modified to 29 items in a health scale, reflecting that most respondents were farmers not in paid employment. The 29 items in final survey were extracted into eight factors, containing different dimensions of social capital.

Our Chinese health version of the Onyx-Bullen scale displayed an acceptable level of internal consistency, with a Cronbach’s α total scale of 0.87, which compares favorably to the original version of the scale where the internal consistency for Onyx-Bullen items was 0.84. Except for *Voluntariness* and *Friends Connections*, all factors displayed fair or good internal consistency with Cronbach’s α greater than 0.5. Although it is difficult to know precisely why *Voluntariness* had relatively low Cronbach’s α, we speculate that there were two possible explanations. First, *Voluntariness* and *Friends Connections* were extracted from the original factors of Onyx-Bullen scale and reclassified into new factors, but with a fewer number of items, which may explain the lower consistency [91]. Second, the items and factors may simply have bad stability and consistency, which we posit is the most likely reason. For *Voluntariness*, there were few volunteer activities or projects attended by villagers in rural China. So voluntary-related questions showed a relatively low applicability in all dimensions of social capital.

The reason for the weak results of *Friends Connections* can be understand by people’s living mode in rural China. Unlike urban places, the Chinese countryside was relatively closed, communicating less with outside villages. People living in rural places have difference social needs from those in cities. The traditional social relationship was based on acquaintance, with people’s main interpersonal relationship based around their neighborhood and relatives, which were geographically close. Rural families had fewer friends than urban families, especially for those rural families with members with chronic diseases [93]. In Raika Abdulahad’s study of Iraqi-Canadians, volunteerism also returned weak results and were excluded from their study [79].

Exploratory factor analysis provided evidence for item attribution. In our study, some items were extracted and some were intergrated. The factor analysis results and comparison with some previous studies are shown in Additional file 1: Table S1. In general, factors representing *Participation in the Local Community, Neighborhood Connections, Proactivity in a Social Context* and *Tolerance of Diversity* were consistent with Onyx and Bullen’s results. Differences mainly appeared in *Feeling of Safety and Trust* and *Value of Life*. Items indicating feelings of safety and feelings of trust were extracted into two factors, which were attributed to one dimension in the original study. Safety and trust were different psychological perceptions in rural China compared to western countries [79]. While there have been discussions of feeling of safety and sense of trust among rural Chinese residents, the relationship between safety and trust have not been discussed together in China [94, 95], except for Xie [96] who concluded there was no significant correlation between interpersonal trust and safety for middle school rural students.

Feelings of trust and self-value were returned to one factor. It is hard to find the consistent explanation in the literature. However, we speculated that this is consistent with the life mode in rural China. As discussed above, compared to urban residents, living in Chinese rural villages meant people had more communication and closer connection with their neighbors [97], but fewer connections outside their village especially for those people with chronic diseases. As a result, for people living in rural villages, most information resources and interpersonal connections were with local village neighbors, including emotional trust and comfort. In Chinese rural communities, the items indicating feeling of trust and value of life loaded into one factor.

In Additional file 1: Table S1, comparisons were made with other studies using the Onyx-Bullen scale. The most consistent factor among all these studies was *Participation in the Local Community,* except for the question “Do you help out a local group as a volunteer?”, which fell into another factor in the current study and was excluded in Abdulahad’s scale on Iraqi-Canadians [79]. The other items showed the same properties under different cultures, which indicates the general nature of the Onyx-Bullen scale and its robustness in different contexts. Factor representing *Feeling of Safety and Trust* presented consistency in two English-version scales, but was split in two language-translated scale studies [79, 81]. In the Abdulahad’s Arabic version scale, items representing feeling of safety were deleted from the total scale because of the race, ethnicity, and religion of the sample. *Social Agency or Proactivity in a Social Context* showed the best consistency with Onyx and Bullen’s study, indicating the items could serve to define this factor well in the poor population with diseases in China. Questions referencing *Neighborhood Connections* and *Family and Friends Connections* were the same in Onyx and Bullen’s and O’Brien’s [80] study, but not in Abdulahad’s or the current study. For the reasons already discussed, *Neighborhood Connections* and *Family and Friends Connections* were extracted into two difference factors in Abdulahad’s and the current study, reflecting country and culture differences.

In order to fully appreciate the current findings, it is important to understand the study limitations. First, the findings were derived from a sample of disease-poor households in Shandong province. However, the results are likely to be generalizable to similar rural populations in China, a proposition that requires further testing. Second, the translation of instruments from one language to another is a methodological challenge for researchers, although such translations are positive and necessary in health research [79]. Although we analyzed a large sample of 802 households, our response rate was 58% due to participants unable to complete the survey due to health or education levels or by declining consent. Surveys in other provinces, other populations, such as urban households, and other rural disease-related poverty households should be undertaken to confirm (or modify) our findings for this rural sample. Finally, this study focused on disease-related poverty people in China, concluding that Onyx and Bullen’s social capital scale had an adequate reliability and validity, but the feasibility about its use in other (sub) population remains to be validated. Social capital dimensions and factors may vary in different culture backgrounds and subpopulation, such as non-Han ethic minority regions with geographic, social and cultural diversity. Our Chinese version of the general Onyx-Bullen scale is available for application to other populations, or to be appropriately modified for other populations. Further work is required to identify the structure and applicability of our Onyx-Bullen health and general scale in other regions and populations in China.

## Conclusion

This paper adapted the Onyx-Bullen social capital scale for a rural subpopulation of disease-poor Chinese rural households, the Chinese Onyx-Bullen health scale. The first step was a forward-backward translation and cultural adaption process to modify the Onyx-Bullen scale for China, deriving the 34 item Chinese Onyx-Bullen general scale. Next, we modified the general Onyx-Bullen scale for a specific rural disease-poor population Chinese population, the 29 item onyx-Bullen health scale. The Chinese Onyx-Bullen health social capital scale showed an adequate reliability and validity when used in a disease-related poverty people in Shandong province. We concluded that the translated Chinese Onyx-Bullen health social capital scale version was a suitable, robust and consistent measurement tool to evaluate social capital for disease-related poverty people in China. We also suggest that further modification of our general and health Onyx-Bullen scale will be required for social capital studies of other populations in China, but our scale provides a platform for any specific changes for other subpopulations in China. With the social capital becoming an increasing important topic in health research, and the social capital condition of poor and diseased households attracting specific research attention, this paper provides a robust instrument to study the social capital status in disease-related poverty households.

## Supplementary information

**Additional file 1: Table S1.**Factor structure of Chinese social capital measure and comparison of factor items.

## Data Availability

The datasets used and/or analysed during the current study are available from the corresponding author on reasonable request.

## Abbreviations

HIV/AIDS: Human Immunodeficiency Virus Infection and Acquired Immune Deficiency Syndrome
SC: Social Capital
