# Supplementary material for: Disease causing poverty: adapting the Onyx and Bullen social capital measurement tool for China
Source: BMC Public Health. 2020 Jan 14;20:63. doi: 10.1186/s12889-020-8163-5 (PMC6961236; doi:10.1186/s12889-020-8163-5)
Supplement: Supplementary file 1 — Additional file 1: Table S1.Factor structure of Chinese social capital measure and comparison of factor items. [file 12889_2020_8163_MOESM1_ESM.pdf]

Table 3 Factor structure of Chinese social capital measure and comparison of factor items

Item	Factors Loading from Rotated Component Matrix								Comparison			
									Current study*	Onyx and Bullen[70]**	Raika Abdulahad[71] ***	O'Brien [72] ****
	1	2	3	4	5	6	7	8	Factor	Factor	Factor	Factor
Do you feel part of the local geographic community where you work?									1	1	1	-
Are your workmates also your friends?									1	1	1	1
Do you feel part of a team at work?									1	1	1	1
At work, do you take the initiative to do what needs to be done even if no one asks you to?									1	5	1	7
In the past week at work, have you helped a workmate even though it was not in your job description?									1	5	1	7
Have you attended a local community event in the past 6 months (e.g., village artistic performance, school concert, craft exhibition)?	.688	.060	.151	.093	.194	.167	-.071	-.047	2	2	2	2
Are you an active member of a local organization or club (e.g., sport, craft, social club)?	.737	.067	.128	.170	.120	.019	.033	-.137	2	2	2	2
Are you on a management committee or organizing committee for any local/village group or organization?	.647	-.102	-.055	.245	-.040	-.146	.133	.163	2	2	2	2
In the past 3 years, have you ever joined a local community action to deal with an emergency?(e.g.,	.728	.011	.044	-.137	.074	.192	-.187	.125	2	2	2	2

emergency office in your village or county)												
In the past 3 years, have you ever taken part in a local community project or working bee? (e.g., labor union)	.794	.006	-.034	-.036	.032	.154	-.067	.086	2	2	2	-
Have you ever been part of a project to organize a new service in your area (e.g., youth club, petition reception offices, children health care center, recreation for disabled)?	.724	.004	.095	.149	.000	-.030	.122	-.056	2	2	2	2
Do you agree that most people can be trusted?	.048	.709	-.032	.138	.124	.038	.099	.100	3	3	3	3
If someone’s car or electric motorcar breaks down (out of power)outside your house, do you invite them into your home to use the phone or charge it?	-.141	.606	.026	.122	.047	.300	.121	.015	3	3	3	-
Does your local community/ village feel like home?	.112	.711	.208	.081	-.147	-.063	.245	.083	3	3	-	3
Can you get help from friends when you need it?	.002	.508	.392	-.091	-.024	.286	.101	.236	3	4	3	5
Do you feel valued by society?	.098	.508	.113	.317	.470	-.021	-.090	-.099	3	7	6	-
If you have only on day left in your life, are you satisfied with what your life has meant?	.005	.621	.016	.215	.362	-.041	-.079	-.099	3	7	6	5
If you were caring for a child and needed to go out for a while, would you ask a neighbor for help?	-.067	.337	.389	.102	-.097	.357	-.037	.378	4	4	3	4
Have you visited a neighbor in the past week?	.152	.043	.579	.284	.030	-.029	.095	.293	4	4	3	4
When you go shopping in your local area are you likely to run into friends and acquaintances?	-.024	.077	.618	.164	.251	.120	.241	-.083	4	4	4	-
In the past 6 months, have you done a favor for neighbors in illness or in need? (including mutual help)	.166	.119	.680	-.048	.024	.092	-.211	.053	4	4	-	4
How many people did you talk to yesterday? 1	.040	.018	.616	.155	.059	.009	.320	.054	4	8	4	8
Do you go outside your local community to visit your family or relatives?	.190	-.018	.172	.473	-.040	.353	-.006	.338	5	5	-	-

If you need information to make a life decision(or an important dicision), do you know where to find that information?	.172	.033	.085	.540	.142	.132	.182	.297	5	5	4	-
If you disagree with what everyone else agreed on, would you feel free to speak out?	.101	.240	.086	.750	.136	-.022	.034	-.006	5	5	7	-
If you have a dispute with your neighbors (e.g., over fences or dogs) are you willing to seek mediation?	.049	.309	.158	.645	.015	.208	-.084	.008	5	5	7	5
Do you think that multiculturalism makes life in your area better?	.132	.053	.174	.065	.777	.104	.120	.203	6	6	5	6
Do you enjoy living among people of different lifestyles?	.149	.118	.026	.076	.812	.141	.077	.170	6	6	5	6
Do you help out a local group or organization as a volunteer?	.234	.198	.033	.017	.104	.602	.133	.097	7	2	-	2
Have you ever help to pick up rubbish in a public place?	.103	.003	.122	.274	.185	.713	.163	-.128	7	5	-	7
Do you feel safe walking down your street after dark?	-.018	.093	.132	.056	.101	.153	.746	-.006	8	3	-	3
Does your area have a reputation for being a safe place?	-.028	.473	.095	-.041	.040	.122	.666	.048	8	3	-	3
In the past week, how many phone conversations have you had with friends and relatives?	.231	.078	.280	.340	.148	-.029	.047	.451	9	8	4	8
How often do you have lunch/dinner with other people outside your household?	-.069	.071	.060	.066	.246	-.008	-.010	.750	9	8	4	8

*Factors in the current study. Factor1=Work Connections; Factor2= Participation in the Local Community; Factor3= Feelings of Trust and Self-value; Factor4= Neighborhood Connections; Factor5= Proactivity in a Social Context; Factor6= Tolerance of Diversity; Factor7= Voluntariness; Factor8= Feelings of Safety; Factor9= Friends Connections.

** Factors in Onyx and Bullen's research. Factor1= Work Connections; Factor2= Participation in the Local Community; Factor3= Feelings of Trust and Safety; Factor4= Neighborhood Connections; Factor5= Social Agency or Proactivity in a Social Context; Factor6= Tolerance of Diversity; Factor7= Value of Life; Factor8= Family and Friends Connections.

*** Factors in Raika Abdulahad's study. Factor1= Work Connections; Facctor2= Participation in the Local Community; Factor3= Family/Friends and Neighborhood Connections; Factor4= Feelings of Trust and Safety; Factor5= Value of Life; Factor6= Tolerance of Diversity; Factor7= Proactivity in a Social Context.

**** Factors in O'Brien's study. Factor1= Work Connections; Factor2= Formal Participation in the Community; Factor3= Feelings of Trust and Safety; Factor4= Neighborhood Connections; Factor5= Value of Life; Factor6= Tolerance of Diversity; Factor7= Proactivity in a Social Context; Factor8= Others.
